# A New Treatment Strategy for Inactivating Algae in Ballast Water Based on Multi-Trial Injections of Chlorine

**DOI:** 10.3390/ijms160613158

**Published:** 2015-06-09

**Authors:** Jinyang Sun, Junsheng Wang, Xinxiang Pan, Haichao Yuan

**Affiliations:** 1 College of Marine Engineering, Dalian Maritime University, Dalian 116026, China; E-Mails: golden_sun@dlmu.edu.cn (J.S.); yuanhc@dlmu.edu.cn (H.Y.); 2 College of Information and Science Technology, Dalian Maritime University, Dalian 116026, China

**Keywords:** ballast water treatment, ion-exchange membrane electrolysis, algal, chlorine, multi-injection

## Abstract

Ships’ ballast water can carry aquatic organisms into foreign ecosystems. In our previous studies, a concept using ion exchange membrane electrolysis to treat ballast water has been proven. In addition to other substantial approaches, a new strategy for inactivating algae is proposed based on the developed ballast water treatment system. In the new strategy, the means of multi-trial injection with small doses of electrolytic products is applied for inactivating algae. To demonstrate the performance of the new strategy, contrast experiments between new strategies and routine processes were conducted. Four algae species including *Chlorella vulgaris*, *Platymonas subcordiformis*, *Prorocentrum micans* and *Karenia mikimotoi* were chosen as samples. The different experimental parameters are studied including the injection times and doses of electrolytic products. Compared with the conventional one trial injection method, mortality rate time (MRT) and available chlorine concentration can be saved up to about 84% and 40%, respectively, under the application of the new strategy. The proposed new approach has great potential in practical ballast water treatment. Furthermore, the strategy is also helpful for deep insight of mechanism of algal tolerance.

## 1. Introduction

The global shipping business carries more than 92% of the world’s commodities; thus, it plays a vital role and massively contributes to world trade. Ballast water is essential to keep the stability of vessels during the voyage from being taken from the source port while no cargo is available, and, to subsequently be discharged at the destination port when the ship takes on cargo [1]. The aquatic organisms may be introduced into foreign ecosystems together with discharged ballast water. Invasive alien species are considered to be one of the greatest threats to biodiversity and have become a national as well as international issue [2]. Organisms mentioned are also related to adverse economical impacts, e.g., destroying marine biodiversity, damaging existing waterways, impacting aquaculture, and causing harmful consequences to human health [3]. The invasion of alien species has become one of the four major threats for the marine ecological environment [4,5]. A ship’s ballast water is one of the main reasons leading to the marine biological invasion worldwide. There are several established and under developed technologies available to be used for ballast water treatment. These techniques are used to mitigate the introduction of non-indigenous aquatic nuisance species from ships’ ballast water. These methods mainly include: physical separation techniques, such as filtration [6] or hydrocyclone separation [7], UV radiation [8], ozonation [9] and chlorination [10], application of biocides as a part of chemical treatment techniques [11], other systems such as sonication [12], and so on. Electrolytic treatment technology is also another alternative method. Electrochemical oxidation has been applied widely in recent years in industrial wastewater, such as textile industry effluents [13,14], as well as in disinfection of drinking, swimming pool, and seawater [15,16]. In our previous studies, we have conducted experiments on the possibility of using ion-exchange membrane electrolytic methods to treat ballast water [17].

For ballast water treatment, the processing time of ballast water treatment should be as fast as possible. Generally, increased ballast water treatment time is one of the main reasons for increasing in-berth waiting time [18]. The time for waiting will unnecessarily put stress on schedule reliability and might even increase miscellaneous items such as port servicing bills [19,20] while the electrolytic oxidative substances will generate disinfection by-products (DBPs) in varying amounts [21]. The toxicological and epidemiological proprieties of disinfection by-products have been investigated in a wide range of studies [22,23]. Most of the research has reported that the disinfection of by-products would affect human health and ecological environment [24].

In order to reduce disinfection by-products dosage and minimize the treatment time, in this paper, a new strategy for inactivating algae is proposed based on the developed ballast water treatment system. In the new strategy, the means of multi-trial injection with small doses of electrolytic products is applied for inactivating algae. To demonstrate the performance of new strategy, contrast experiments between new strategies and routine process were conducted.

## 2. Results and Discussion

### 2.1. One Trial Injection of Electrolytic Products

**Figure 1:**
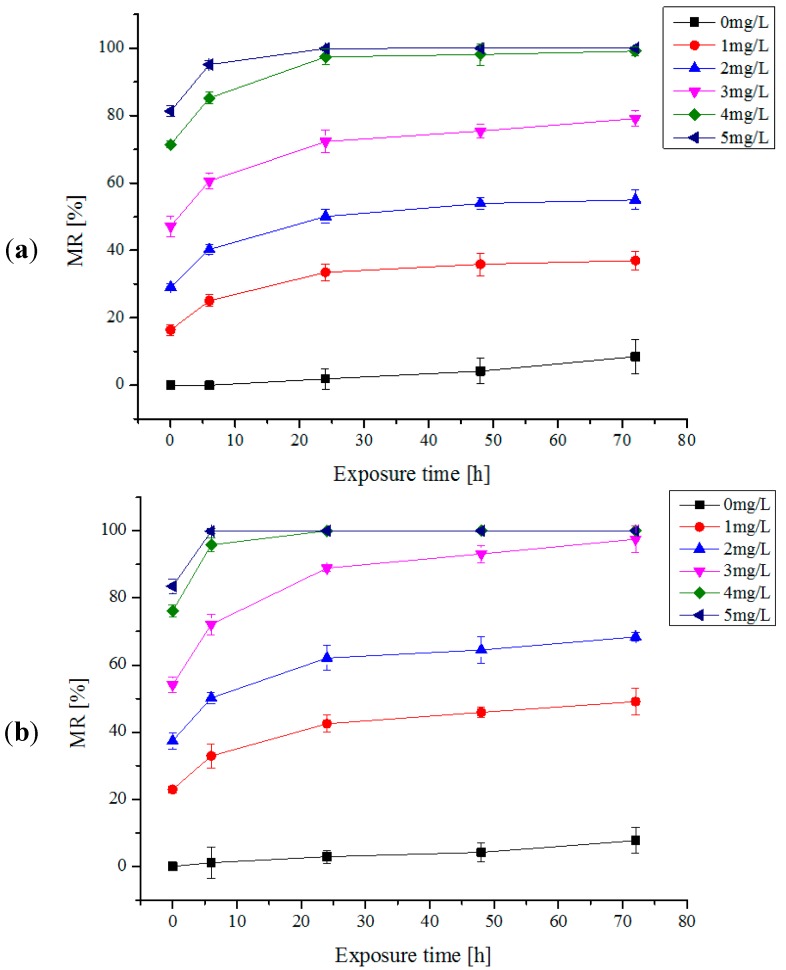

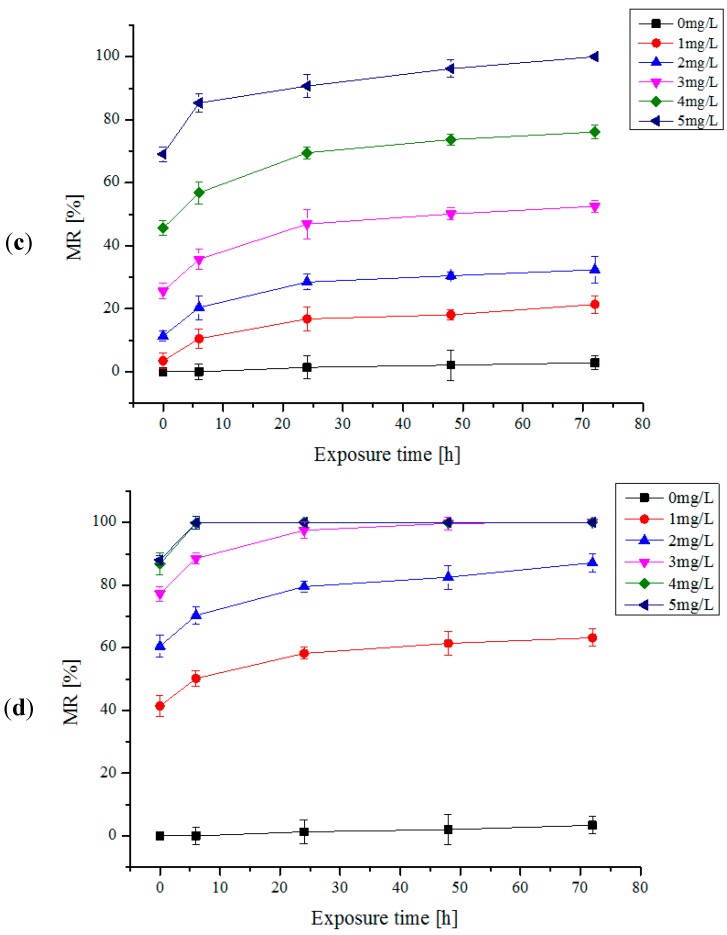
(**a**) The mortality rates (MRs) of *Platymonas subcordiformis* by one trial injection of electrolytic products; (**b**) The MRs of *Chlorella vulgaris* by one trial injection of electrolytic products; (**c**) The MRs of *Prorocentrum micans* by one trial injection of electrolytic products; (**d**) The MRs of *Karenia mikimotoi* by one trial injection of electrolytic products.

**Figure 2:**
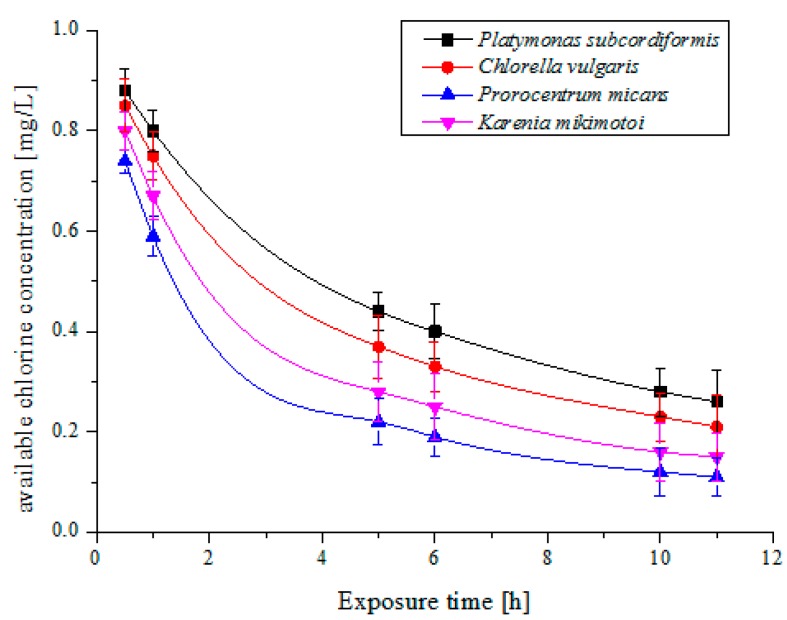
The decay curve of 1 mg/L available chlorine concentration with time for the four microalgae species.

The mortality rates (MRs) of algae species by one trial injection of electrolytic products are shown in Figure 1a–d. The relations among the algae mortality and the electrolytic products concentration and the reaction time were illustrated in the figures. It can be seen that with the increase of the concentration of the available chlorine, the MRs of four algae species increase. When the concentration of available chlorine reaches 5 mg/L, all algae species died completely after 72 h. It is observed that the trends of the relations among the MR and the electrolytic products concentration and the reaction time for all algal cells are almost the same. However, the tolerance ability of each algae species against available chlorine is quite different. For *Platymonas subcordiformis*, when the concentration of available chlorine was 5 mg/L, the MR reaches 100% after being treated for 24 h, *Chlorella vulgaris*, 4 mg/L for 100% after 24 h, *Karenia mikimotoi*, 3 mg/L for 100% after 72 h, and *Prorocentrum micans*, 5 mg/L for 100% after 72 h respectively. In addition, the MRs of algae species is related also to exposure time of available chlorine. Under the certain concentrations of the available chlorine, the MR of algae increases with the exposure time. It is found that the MRs of algal cells rise significantly within 6 h. While under extended exposure time, the MRs of algal cells gradually decreased. This phenomenon, in turn, is related to available chlorine decay or deterioration [25]. Typically, the decay characteristics of 1 mg/L available chlorine concentration with the exposure time for the four algae species were shown in Figure 2.

### 2.2. Multi-Trial Injection with a Small Dose of Electrolytic Products

**Figure 3:**
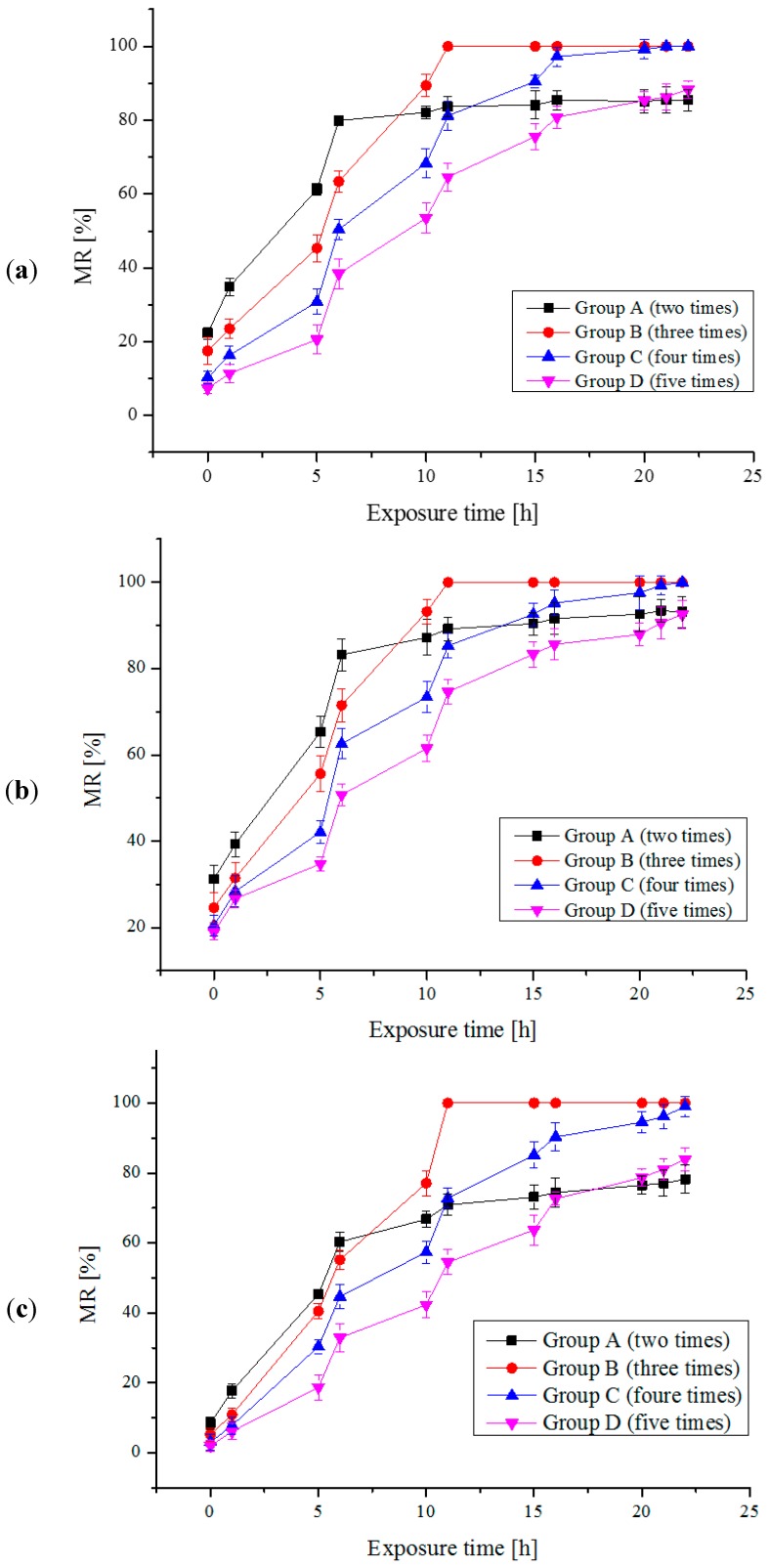

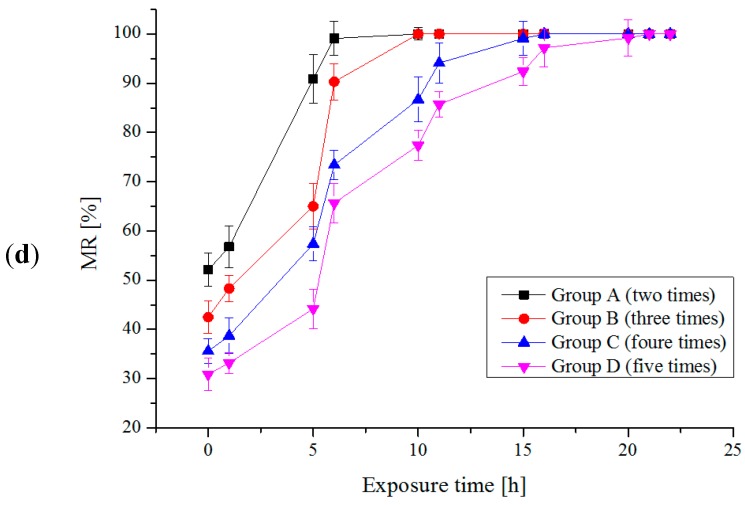
(**a**) The MRs of *Platymonas subcordiformis* by multi-trial injection with a small dose of electrolytic products; (**b**) The MRs of *Chlorella vulgaris* by multi-trial injection with small dose of electrolytic products; (**c**) The MRs of *Prorocentrum micans* by multi-trial injection with small dose of electrolytic products; (**d**) The MRs of *Karenia mikimotoi* by multi-trial injection with small dose of electrolytic products.

As mentioned above, four groups of different available chlorine concentrations and injection times were used to treat each algal species, which are Group A (2 times, 1.5 mg/L), Group B (3 times, 1 mg/L), Group C (4 times, 0.75 mg/L), Group D (5 times, 0.6 mg/L), respectively. The interval time between two times is 5 h. The MRs of the four algae species are shown separately in Figure 3a–d. We can see that MRs increase with the exposure time for each algae species under the certain available chlorine concentration. It should be noticed that the needed exposure time are different for different treatment groups when MRs reach the same value; in other words, the speed of MR increase of different treatment strategies are different. Among these four treatment strategy groups, the required exposure time of Group B is shortest when MR reaches 100%. Therefore, Group B (3 times, 1 mg/L) is chosen as an optimal strategy to treat the algal cells. Comparison between the treatment strategy of Group B and the conventional one trial injection method were conducted. The experimental results are shown in Table 1. Compared with the conventional one trial injection method, mortality rate time (MRT) and total available chlorine dose can be saved up to about 84% and 40%, respectively, under the application of the new strategy. In addition, the investigation of algal resurrection was also carried out. There is no algal resurrection phenomenon for all of the killed algal cells (MR is 100%) within 15 days after being killed.

**Table 1:** Comparison between the treatment strategy of Group B and conventional one trial injection treatment methods.

Species	Available Chlorine Concentration (mg/L)		Exposure Time (h)		Results	
	Conventional Experiment	Strategy Method	Conventional Experiment	Strategy Method	Saving Dose	Saving Time
*Platymonas subcordiformis*	5	3	48	11	40%	77%
*Chlorella vulgaris*	4	3	24	11	25%	54%
*Prorocentrum micans*	5	3	72	11	40%	84%
*Karenia mikimotoi*	4	3	24	11	25%	54%

### 2.3. Discussion

**Figure 4:**
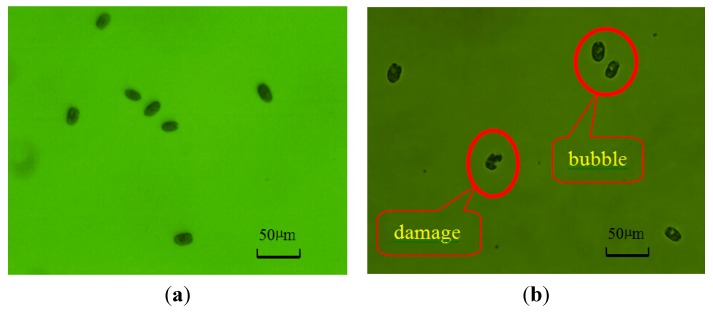
(**a**) Before being treated; (**b**) After being treated. The appearance images of the algal cells (*Platymonas subcordiformis*) before and after being treated by using the developed ion exchange membrane electrolysis system.

Experimentally, the effectiveness of the strategy of using multiple injections with small doses to treat algal cells has been demonstrated. The possible mechanisms were discussed as follows:

Firstly, in ion exchange membrane electrolysis system, hypochlorous acid molecule is a key composition in available chlorine and would penetrate and damage gradually membrane of algal cells and enter the cytoplasm in algal cells [26,27,28]. It would cause the release of intracellular organic matter (IOM) and also increase the levels of dissolved organic matter (DOM). These substances would lead to the extensive damage of cell membranes and the resulting changes of external cell architecture until algal cells die [29,30]. Figure 4 shows the appearance images of the algal cells (*Platymonas subcordiformis*) before and after being treated by using the developed ion exchange membrane electrolysis system. Before being treated, the algal cells have spherical shapes and smooth surfaces. However, after being treated, the membranes were damaged and have some bubbles in cells.

The tolerance of algal cells to hypochlorous acid molecules means the defense ability of the membranes of algal cells against extrusion and damage of hypochlorous acid molecules [31,32]. The constant shocks upon membranes of algal cells brought by multiple injections may cause the decline of defense ability of membrane of algal cells, and further accelerate the rate of membrane damage. Thus, it would need less available chlorine concentration and the shorter exposure time to make algal cells dead completely. It may be a reason why the needed exposure time in Group B (three times) is shorter than that in Group A (two times), which is much shorter than that in the one trial injection mode when MR reaches 100%.

On the other hand, available chlorine concentration in algal solutions is not constant and would decay gradually with time and some of them may have disappeared before they could penetrate into a cytoplasm of algal cells for a one trial injection. When the available chlorine concentration decreases to a certain value, the membrane of algal cells cannot be penetrated any more. In this situation, higher available chlorine concentration and longer exposure time would be necessary and inevitable. Nevertheless, on the other hand, multiple injections could keep the available chlorine concentration to a stable level which may be more than the threshold and thus easier to penetrate the membrane of algal cells. Thus the utilization ratio of total available chlorine concentrations in multiple-injection mode would be higher than that in one trial injection condition. It may be a key reason why the needed exposure time in Group B (1 mg/L) is shorter than that in Group C (0.75 mg/L), which is shorter than that in Group D (0.6 mg/L) which is much shorter than that in one trial injection mode when MR reaches 100%.

## 3. Experimental Section

### 3.1. Principle of Ion Exchange Membrane Electrolysis

**Figure 5:**
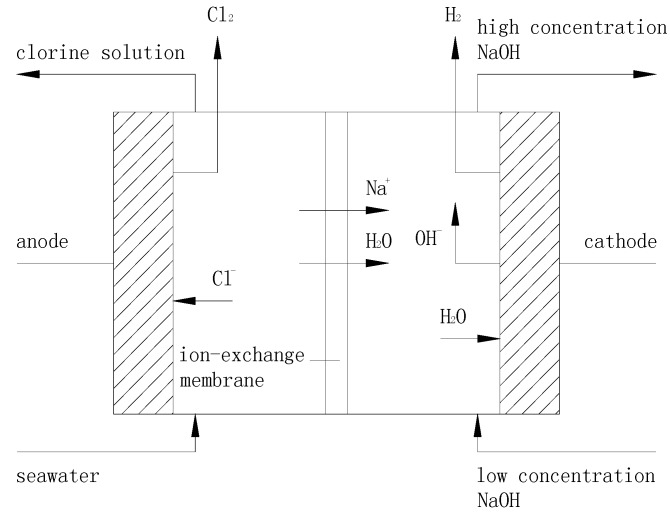
The operating principle of ion-exchange membrane.

The operating principle of ion exchange membrane electrolysis is shown in Figure 5. The electrolytic anode chamber and cathode chamber are separated by ion-exchange membrane. The products of the electrolytic anode are used for the ballast water treatment and products of the electrolytic cathode are used for the flue gas desulfurization treatment.

The ion-exchange membrane is a perfluorinated cation exchange membrane which is a special kind of cationic selective permeable membrane. The membrane allows cations and water molecules to go through the membrane where anion and gas molecules are prohibited. After electrolysis, chlorine solution is produced as anode products. The composition of the chorine solution includes HCl, HClO, NaClO_3_, Cl_2_, ClO^−^, Cl^−^, [O] *etc.*, in which HClO was used for treating algae in ballast water. The anode reaction that occurs in the ion-exchange membrane electolyzer can be depicted as:

2Cl^−^ → Cl_2_↑ + 2e (1)

Cl_2_ + H_2_O → HCl + HClO (2)

### 3.2. Setup and Procedures of the Ion Exchange Membrane Electrolysis System

**Figure 6:**
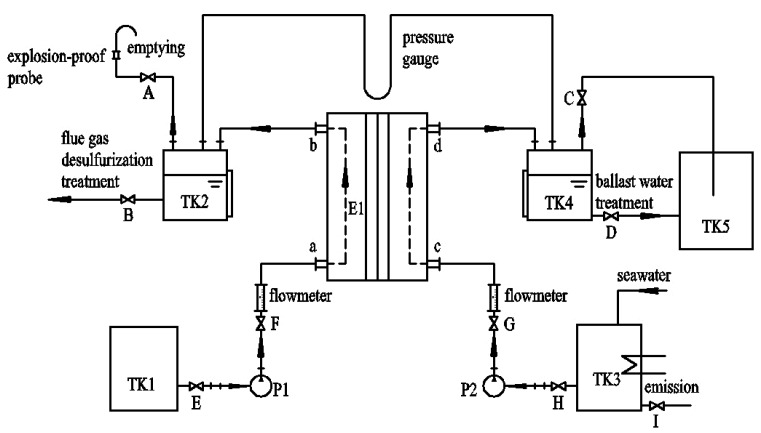
Schematic diagram of the ballast water treatment system. E1—electrolyzer; TK1—low concentration lye tank; TK2—high concentration lye tank; TK3—seawater tank; TK4—gas-liquid separator tank; TK5—Erlenmeyer flask; P1 P2—seawater pump; A–I—stop valve, a,c—inlet, b,d—outlet.

Based on the above-mentioned principle of ion exchange membrane electrolysis, a new ballast water treatment system is presented in this paper. The schematic diagram of the presented ion-exchange membrane electrolysis system is shown in Figure 6. It is mainly composed of 14 parts which are: electrolyzers, magnetic drive pumps, magnetic circulation pumps, water storage tanks, cathode gas-liquid separator tanks, brine circulation tanks, anode gas-liquid separation tanks, glass rotor flowmeters, U-shaped pressure gauges, temperature control instruments, high frequency direct current (DC) power supplies, electric heaters, pressure gauges and thermometers, respectively. The details of the components in the treatment system were shown in Table 2. Seawater will be pumped in the electrolyzer from the bottom of electrolytic anode compartment (position c in Figure 6). The solution containing a low concentration of NaOH is pumped in the electrolyzer from the bottom of the cathode compartment (position a in Figure 6). After electrolysis, the available chlorine solution flows from the top of the anode compartment (position d in Figure 6) to the ballast water tank. The chlorine solution is used for ballast water treatment. The product of electrolytic cathode (high concentration NaOH) outflows from the top of the cathode compartment (position b in Figure 6) to the high concentration lye tank. The concentration of NaOH solution used in the experiments was 15%, the flowrate of the low concentration NaOH solution was 40 L/h, the temperature at which the electrolysis took place was 75 °C. The Erlenmeyer flasks (500 mL/each) were used to simulate the ballast water tank, where the experiments of the microalgae treatments were conducted. Inactivation algal experiments are carried out when the electrolyzer is under the optimum operating condition. Referring to experiment results, the current density of 1.5 KA/m^2^ is found to be the best while the system is working under mentioned parameters. In addition, corresponding current 300 A, current efficiency 80%, cell voltage 4.27 V, available chlorine concentration 800 mg/L are also applied.

**Table 2:** The main components of the treatment system.

No.	Equipment Name	Size	Quantity
1	Electrolyzer	400 × 500 × 110 (mm)	1
2	Magnetic drive pump	3 m; 15 L/min	2
3	Magnetic circulation pump	2.1 m; 5.5 L/min	1
4	Water storage tank	Stainless steel 720 × 500 × 600 (mm)	1
5	Cathode gas-liquid separator tank	Stainless steel 700 × 500 × 600 (mm)	1
6	Brine circulation tank	φ-600; h-600 (mm)	1
7	Anode gas-liquid separation tank	φ-600; h-600 (mm)	1
8	Glass rotor flowmeter	6–60 L/h	2
9	U-shaped pressure gauge	5000 Pa	1
10	Temperature control instrument	−49.9–149.9 °C	2
11	High frequency dc power supply	380 V × 3	1
12	Electric heater	220 V; 2 kW	2
13	Pressure gauge	0–0.25 Pa	1
14	Thermometer	0–100 °C	1

### 3.3. Algae Species Preparation

Strains of algae, namely, *Chlorella vulgaris*, *Platymonas subcordiformis*, *Prorocentrum micans* and *Karenia mikimotoi* are obtained from the Marine Fisheries Research Institute of Liaoning Province, China. The algae species were cultured by CO_2_ Light incubator (MGC-300A, Shanghai YiHeng, Shanghai, China) and they were cultivated by *f*/2 medium [33]. The bottles are shaken four times at regular intervals every day. The illumination was 3000 lx while the light and dark cycle was 12 h:12 h. The seawater in the experiment was prepared by dark precipitate, sand filtered, and boiled for disinfection before being used. The salinity was 31, pH 8.0 and the temperature was 25 °C.

### 3.4. Algae Inactivation Evaluation and Treatments

#### 3.4.1. Algae Inactivation Evaluation

Mortality rate (MR) is used to evaluate the performance of the inactivation treatment. Mortality is the proportion of the dead algal cell number over the total cell number, which is defined by Equation (3):

(3) $$\mathrm{MR}=\frac{\mathrm{number}\mathrm{of}\mathrm{death}\mathrm{algae}\mathrm{cells}}{\mathrm{total}\mathrm{algae}\mathrm{cells}}\times \mathrm{100\%}$$

The evaluation of MR can be accomplished by using flow cytometry (FACSCalibur™ Cytometer, BD Biosciences, Franklin Lake, NJ, USA) [34]. Mortality rate time (MRT) means the required time when MR reaches 100%.

#### 3.4.2. Inactivation Algae Test by One Trial Injection of Electrolytic Products

The initial concentrations of *Chlorella vulgaris* and *Platymonas subcordiformis* were 1 × 10^5^ cell/mL while the initial concentrations of *Prorocentrum micans* and *Karenia mikimotoi* were 1 × 10^3^ cell/mL. Different concentrations of electrolytic products were injected into each algae species bottles, and the available chlorine concentration of each algal solution were 1, 2, 3, 4, 5 mg/L respectively. After being treated, the algal cells in the Erlenmeyer flask were shaken evenly and were then taken out by a pipette. Every experiment was repeated three times. The mortality of algae was determined at 0, 6, 24, 48, 72 h, respectively.

#### 3.4.3. Inactivation Algae Test by Multi-Trial Injection with Small Dose of Electrolytic Products

The initial concentrations of the algal samples and the means of algal sampling are same to those in the one trial injection (shown in Section 3.4.2.). The injection mode of electrolytic products was changed for the new strategy in which electrolytic products are respectively injected two times (Group A), three times (Group B), four times (Group C), and five times (Group D) accordingly. Available chlorine with concentration of 1.5 mg/L is injected for Group A. For Group B, available chlorine with concentration of 1 mg/L is injected. Group C and D are injected with concentration of 0.75 and 0.6 mg/L respectively. As a result, the total injection concentration is 3 mg/L for each group. Electrolytic products are injected at every five hours interval. The mortality of algae was determined at 0, 1, 5, 6, 10, 11, 15, 16, 20, 21 and 22 h, respectively.

## 4. Conclusions

A new strategy of multiple injections with small doses is introduced in this paper. The effectiveness of the new strategy was demonstrated experimentally. Compared with the conventional one trial injection method, the new strategy can greatly reduce by-product doses and exposure time in ballast water treatment, and mortality rate time (MRT) and available chlorine concentration can be saved up to about 84% and 40%, respectively, under the application of new strategy. The proposed new approach has great potential in practical ballast water treatment. Furthermore, the strategy is also helpful for deep insight of mechanisms of algal tolerance.
